# CRISPR CLIP: comprehensive reviews on interventional studies using precision recombinant technologies: clinical landmarks, implications, and prospects

**DOI:** 10.1093/oxfimm/iqae013

**Published:** 2024-11-21

**Authors:** Swarali Yatin Chodnekar, Zurab Tsetskhladze

**Affiliations:** Faculty of Medicine, University Geomedi, 3, King Solomon Street, Tbilisi 0114, Georgia; Faculty of Medicine, University Geomedi, 3, King Solomon Street, Tbilisi 0114, Georgia

**Keywords:** CRISPR-Cas9, genome editing, therapeutics, cancer, genetic diseases, hematological diseases, infections

## Abstract

To consolidate clinical trials that utilized the CRISPR technology to synthesise cures for various genetic diseases as a means to provide a window into the progress made so far while paving the way forward for future research and practices. Systematic review (PROSPERO CRD42023479511). Trials from seven databases’ (ClinicalTrials.gov, European Union Clinical Trials Registry, ISRCTN registry, ICTRP/trialsearch.who.int, ChiCTR.org.cn, Clinical Trial Registry India, and Cochrane Library/Trials) inception to 9 March 2024, were considered. Exclusion criteria were unrelated, duplicated, non-English, unavailable full texts, diagnostic studies, correlational studies, observational studies, abstract-only papers, reviews or conference papers. Included studies were appraised using the ten-item CASP tool to assess methodological quality. The review identified 82 RCTs utilizing CRISPR and revealed four main themes: Diseases targeted, Countries of Clinical trials, Type of interventions, and Trial trends over the years. Geographically, the United States and China lead in the number of CRISPR clinical trials, followed by the European Union. However, Africa, Asia, and South America have very few trials. Among disease classes, cancer is the most prevalent focus with 39 studies, followed by monogenetic blood diseases, like Thalassemia and sickle cell anaemia. The biological agent CTX001 and Cyclophosphamide each feature in 11 studies. The peak year for clinical trials was 2018, marked by a significant increase with 16 studies conducted. Despite conducting a comprehensive search, the majority of trials were concentrated in the United States and China. Additionally, potential oversights due to vague titles, English-only studies, and indexing issues may have occurred. Nonetheless, by incorporating data from seven distinct databases, this review significantly contributes to understanding CRISPR's utilization in therapeutic clinical trials, paving the way for future research directions. The review underscores the burgeoning interest in CRISPR-based interventions. Current trials barely tap CRISPR's potential for treating genetic diseases.

## Introduction

Genetic engineering is a technology in which DNA is inserted, deleted, modified or replaced on a cellular level in the genome. Gene editing has enabled us to create suitable models of pathological processes. These modifications are sequence specific and can be implemented on a broad spectrum of organisms and cell types.

In the recent past, genetic modification of mammalian cell lines was considered to be financially exhaustive, laborious and highly restricted to specialized laboratories [1]. Yet today, due to the availability of genome editing technologies like CRISPR-Cas9, the process has become user-friendly and cost effective. Scientists are now able to customize any genetic information and create new cell lines within a matter of weeks [2].

Therefore, there is an urgent need to investigate the predominant technologies for genome editing in human therapeutics. The purpose of this systematic review was to consolidate clinical trials that use CRISPR-Cas9 for curing Human Diseases and to gain a deeper and more holistic insight into their mechanisms and to pave a way forward by providing future potentials and limitations—with the objective of tackling these eventualities.

## Materials and methods

### Aim

This systematic review aimed to synthesize knowledge on the clinical trials that use CRISPR gene editing technology for curing Human diseases and quantify the data on the geography, demographics, modes of intervention and the diseases for which the cures were generated.

### Design

Summarize and synthesise evidence from the included clinical trials.

Our research questions included:

- What are the applications and results of using CRISPR and gene editing technologies in treating genetic diseases?
- What are the future implications and potential pitfalls to CRISPR?

### Search strategy and screening

This review adopted the PRISMA (The Preferred Reporting Items for Systematic Reviews and Meta-Analyses) guidelines [3]. The PRISMA checklist can be referred to in the supplemental material 3 and 4. The research protocol was registered on PROSPERO (PROSPERO CRD42023479511).

A systematic search of electronic databases was used to identify the studies.

For relevance and validity purposes, seven electronic databases (ClinicalTrials.gov, European Union Clinical Trials Registry, ISRCTN registry, International Clinical Trials Registry Platform (ICTRP), ChiCTR.org.cn, trialsearch.who.int and Cochrane Library/Trials) were searched and sorted on the basis of their relevance and availabilities. The search database included English-only studies until 9 March 2024.

The titles and abstracts of the studies were screened prior to full-text screening according to the eligibility criteria. The search strategy for the seven databases can be found in Supplementary Table S1. Any discrepancies that arose were discussed between the authors until a consensus was reached.

### Eligibility criteria

Criteria for including studies in the review

- Population: People with any disease resulting from genetic anomaly; any age, any gender and any severity.
- Interventions: CRISPR genome editing technology.
- Comparisons or control groups:
- Outcomes of interest: Prevalence of CRISPR supported techniques used for curing genetic disease,
- Setting: Hospital admissions/secondary care
- Study designs: Clinical Trials

Exclusion criteria were unrelated, duplicated, non-English, unavailable full texts, diagnostic studies, correlational studies, observational studies, abstract-only papers, reviews or conference papers.

### Critical appraisal

The included studies were appraised critically using a ten-item CASP (Critical Appraisal Skills Programme) tool (https://casp-uk.net/casp-tools-checklists/). The studies were assessed for the sampling strategies, methodologies, designs, values of the research, ethical considerations, clarity of their aims, rigors of data analyses, statements of the findings, reflexivity of the researchers, and data collections. Most of the studies were in the phase of recruiting or ongoing interventions. This did not affect the outcomes of this Systematic Review as it aims to quantify absolute data trends. The exceptions to these were the following clinical trials: NCT04244656, NCT04767308, NCT03655678, NCT03745287, NCT04601051, NCT04502446, NCT02793856, NCT04557436, NCT05397184, NCT04178382, NCT03545815, NCT03164135, NCT03872479. These trials have concluded and have their results published. Overall, most trials demonstrated robustness and had neutral to positive evaluations for all the questions in the CASP tool.

### Data extraction

According to the PRISMA checklist, the data was extracted in two steps [4]. Firstly, publication details (Study Title, Study Type, Publication Number, Conditions, Interventions, Study Design, Country) and findings were extracted for a subsequent thematic analysis.

Quantification of the data was carried out for the following themes:

- Diseases targeted,
- Geographical Distribution of Clinical trials,
- Type of interventions, and
- Trial trends over the years.

Any discrepancies that arose were discussed between the authors until a consensus was reached.

## Results

### Search outcomes

**Figure 1.**
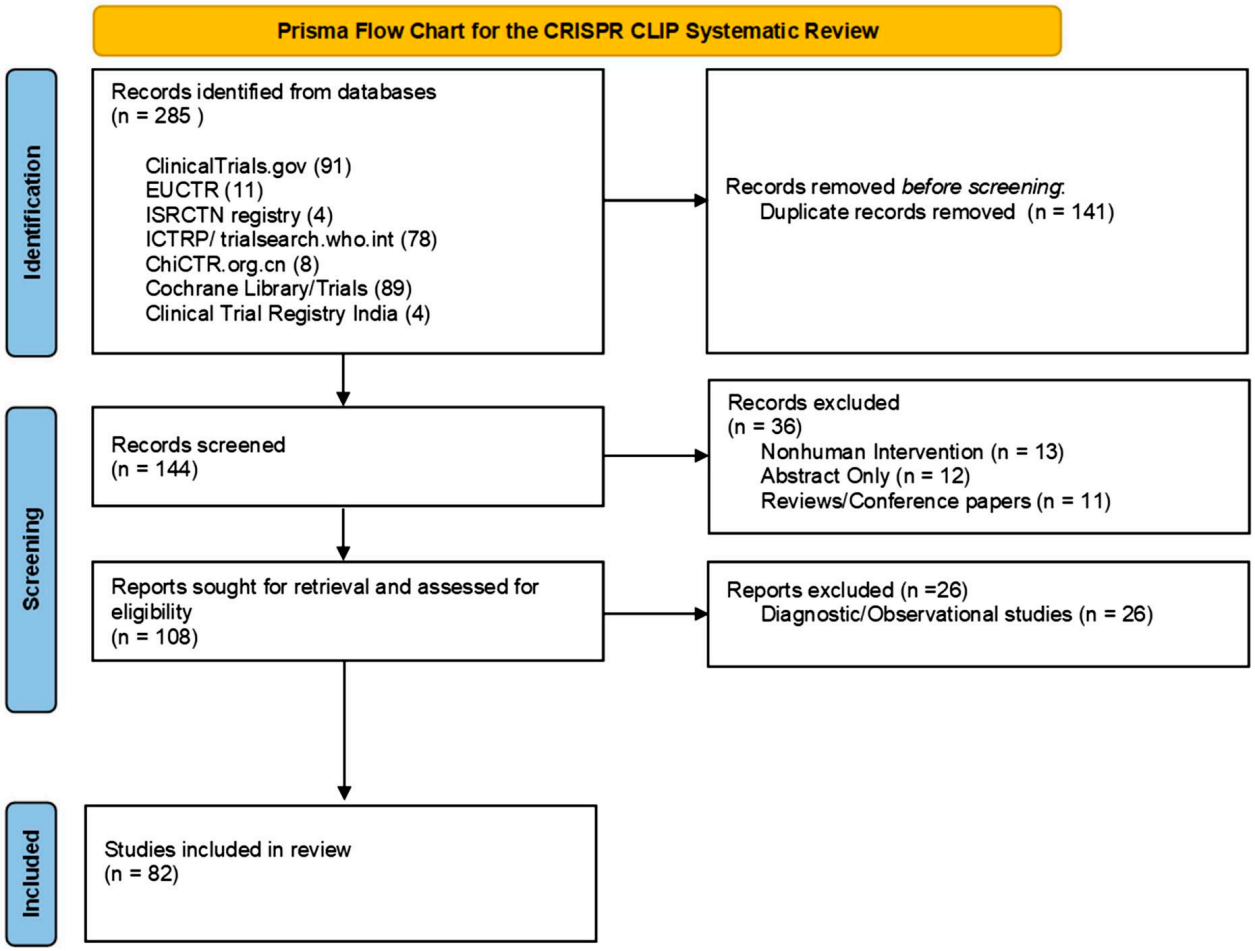
PRISMA flowchart for study screening and inclusion for the present review.

Two hundred and eighty five studies were initially identified, and 144 studies were screened for their titles and abstracts after the removal of 141 duplicates. One hundred eight studies were retrieved for full-text reviews, after 36 studies were excluded as they did not fulfil the eligibility criteria. Twenty-six studies were deemed irrelevant based on study designs. The details of these 26 studies can be found in Supplementary Table S2. The process led to the inclusion of 82 studies in this review [5–86]. Figure 1 shows the PRISMA flow diagram. The details of the 82 included studies can be found in Supplementary Table S1.

### Characteristics of the studies

This study examined all the clinical trials in which gene editing has been utilized in a therapeutic capacity. Here four trends emerged as listed below.

#### Study distribution by geography

**Figure 2.**
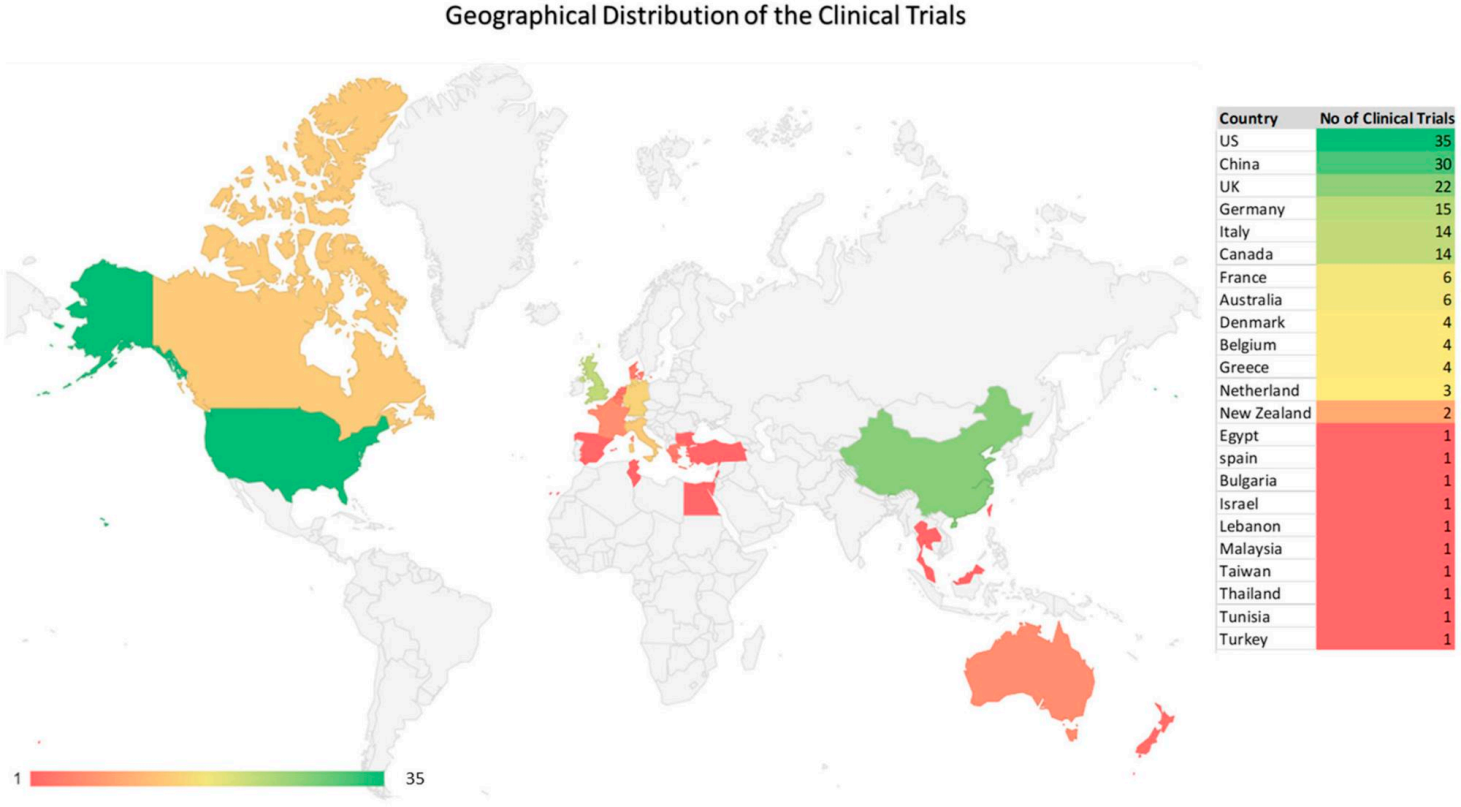
Geographical Distribution for the clinical trials.

As demonstrated in Fig. 2, in the global clinical trial distribution, the USA leads with 35 trials, followed by China with 30. The UK with 22. Germany and Italy each have 15 and 14 trials, respectively, while Canada hosts 14. France and Australia each contribute 6 trials. Denmark and Belgium have 4 each, and Greece, the Netherlands, and New Zealand have 4, 3, and 2 trials, respectively. Egypt, Spain, Bulgaria, Israel, Lebanon, Malaysia, Taiwan, Thailand, Tunisia, and Turkey each have 1 trial.

#### Study distribution according to the disease classes

Cancer leads with 39 trials, followed by Monogenetic blood diseases 24, particularly Thalassemia, with 13 studies. Infections and Sickle cell anaemia are each represented by 10 and 12 trials, respectively. Additionally, diabetes, Inherited retinal dystrophies (NCT03872479, NCT04560790) and angioedema (EUCTR2021-001693-33, NCT05120830) each have two trials, while singular trials are allocated to Transthyretin-Related (ATTR) Familial Amyloid Disease (NCT04601051), and Duchenne Muscular dystrophy (NCT05514249).

Infectious Diseases encompass 1 trial for urinary tract infections due to E. coli (EMBASE 637443513), 3 studies for HIV-1 infection (NCT03164135, NCT05143307, NCT05144386), and singular studies for COVID-19 Respiratory Infection (NCT04990557) and Severe Pneumonia (NCT05143593).

**Figure 3.**
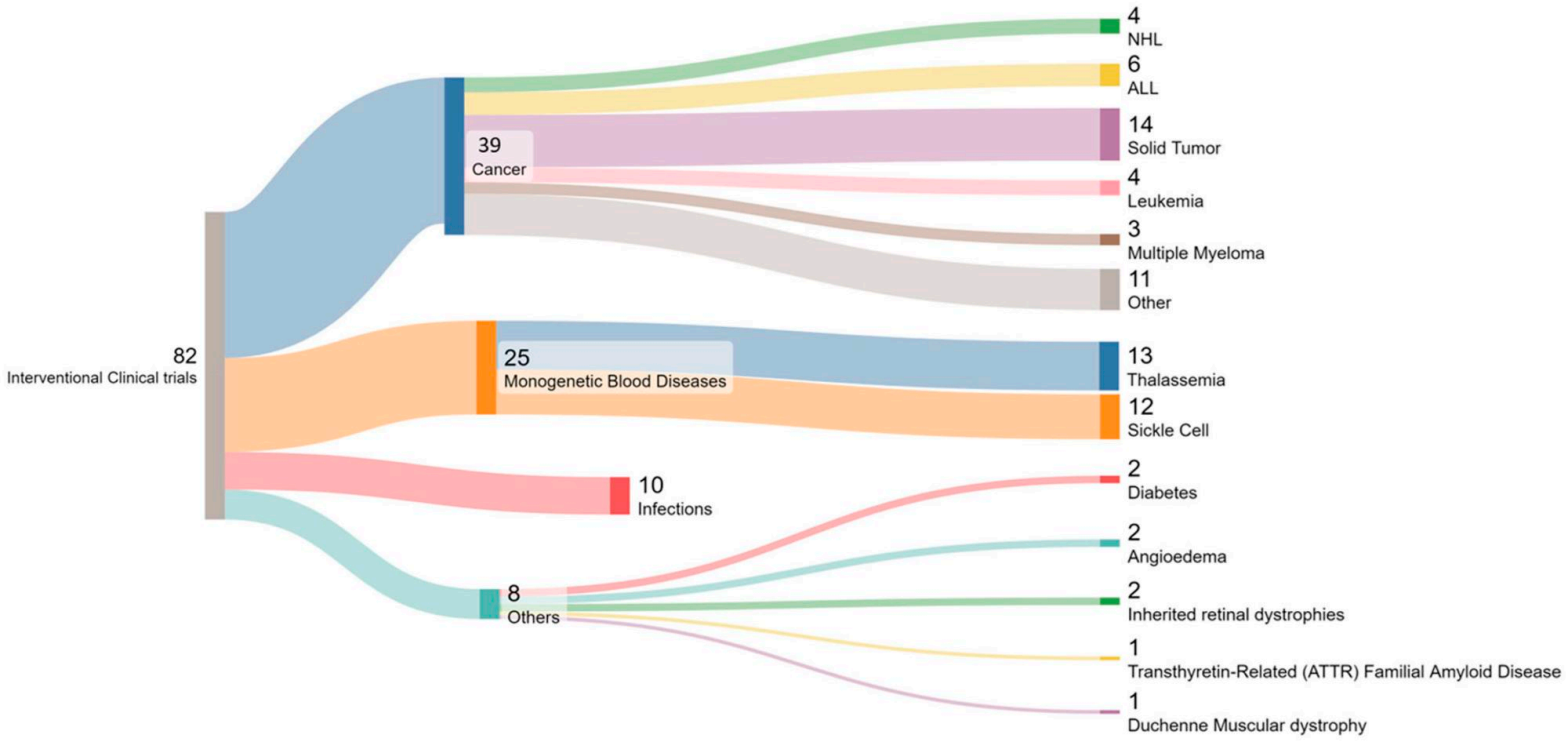
Sankey Diagram for the visualization of the distribution of trials across various diseases.

In the domain of Cancers/Tumours, each specific type is dedicated to 1 study, including Metastatic Non-small Cell Lung Cancer, Invasive Bladder Cancer Stage IV, Metastatic Renal Cell Carcinoma, Stage IV Gastric Carcinoma, Oesophageal Cancer, and Gastrointestinal Epithelial Cancer. Blood-related Cancers are studied extensively, such as B-cell Acute Lymphoid Leukaemia (B-ALL) in 3 studies, Relapsed/Refractory B-cell Acute Lymphoid Leukaemia in 1 study, T cell leukaemia in 1 study, and Chronic Lymphocytic Leukaemia (CLL) in 1 study. Non-Hodgkin Lymphoma and its subtypes have 5 studies, while T-Cell Lymphoma, B-cell Lymphoma, and Multiple Myeloma are the focus of 2, 3 and 2 studies respectively. Furthermore, there is 1 study each for Advanced Hepatocellular Carcinoma, Pancreatic Neoplasms, Prostate Cancer, Human Papillomavirus-Related Malignant Neoplasm, and 3 trials for Solid Tumours. Refer Figure 3.

#### Study distribution by intervention

Biological CTX001 has been utilized in 11 studies (EUCTR2017-003351-38-DE, EUCTR2017-003351-38-GB, EUCTR2018-001320-19-BE, EUCTR2018-002935-88-GB, EUCTR2021-002172-39-DE, EUCTR2021-002173-26-DE, NCT03655678, NCT03745287, NCT05329649, NCT05356195, NCT05477563).

Drug Cyclophosphamide showcases a broad application and has been featured in 11 studies, addressing conditions such as Metastatic Non-small Cell Lung Cancer, Invasive Bladder Cancer Stage IV, Metastatic Renal Cell Carcinoma, Stage IV Gastric Carcinoma, Gastrointestinal Epithelial Cancer, B-cell Acute Lymphoid Leukaemia (B-ALL), Chronic Lymphocytic Leukaemia (CLL), and Multiple Myeloma (NCT02793856, NCT02863913, NCT02867332, NCT03044743, NCT04037566, NCT04767308, NCT03399448, NCT04426669, NCT04637763, NCT05566223, NCT05631912).

Meanwhile, Drug Fludarabine has been part of eight trials, focusing on Stage IV Gastric Carcinoma, Gastrointestinal Epithelial Cancer, B-cell Acute Lymphoid Leukaemia (B-ALL), T-Cell Lymphoma, and Multiple Myeloma (NCT03044743, NCT04037566, NCT04767308, NCT03399448, NCT04426669, NCT04637763, NCT05566223, NCT05631912).

Lastly, the Genetic/CRISPR-Cas9 CTX110 intervention has been applied in two studies related to blood-related cancers, specifically addressing Non-Hodgkin Lymphoma and B-cell Lymphoma (EUCTR2018-003916-38-DE, NCT04035434).

#### Trial distribution by years

**Figure 4.**
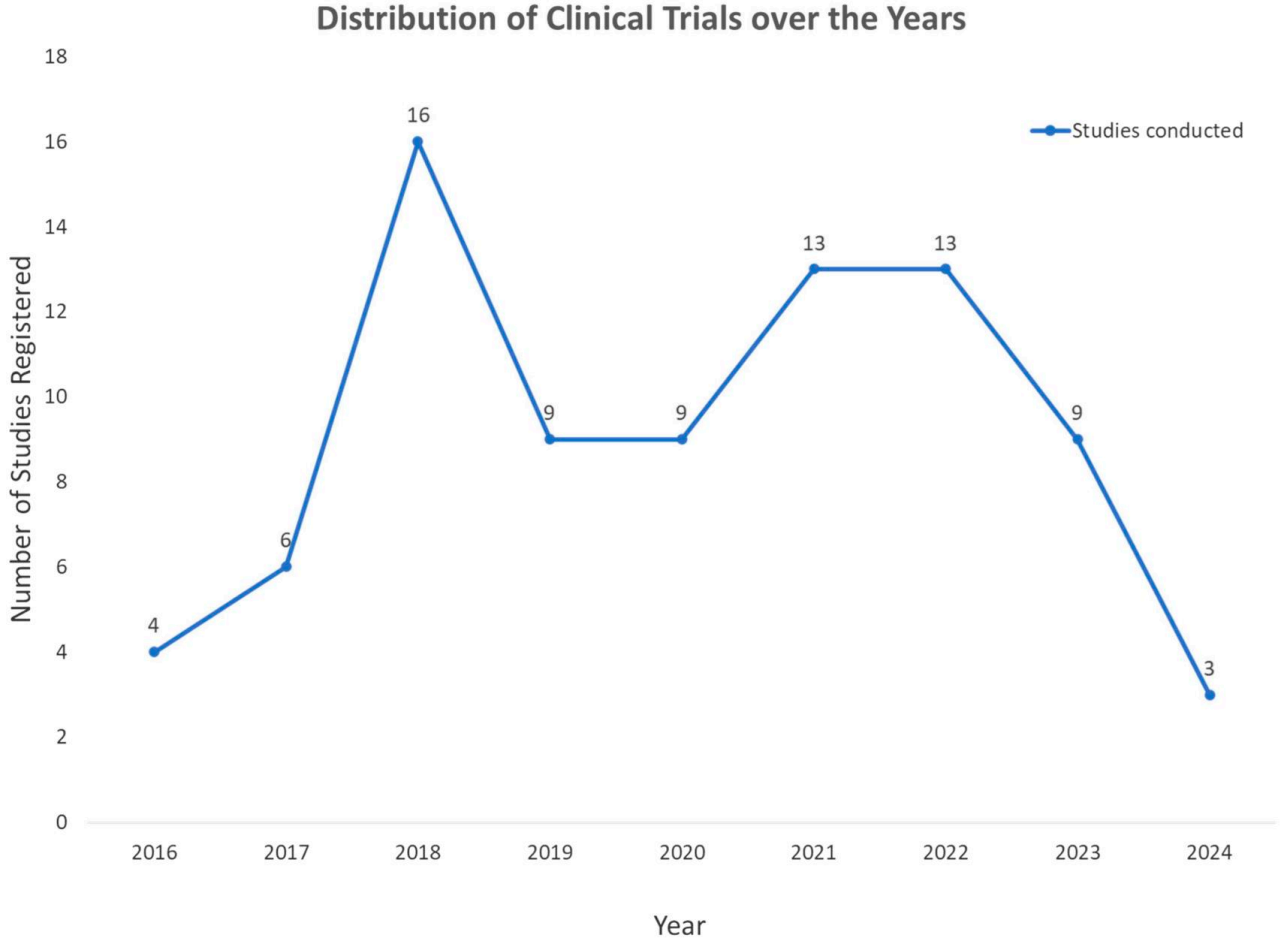
Graph of the distribution of CRISPR trials over the years.

In 2016, four studies were conducted. The number increased to six in 2017. Subsequently, in 2018, there was a notable surge with 16 studies. In 2019, the count decreased to nine, maintaining this level in 2020. In 2021, there was a resurgence, with 13 studies, a figure sustained in 2022. The number of studies registered for 2023 was nine, and for the year 2024, three studies have been registered. Refer Fig. 4.

### Risk of bias

This systematic review has a low risk of bias due to its robust methodological framework:

- **PRISMA Guidelines and PROSPERO Registration**: Adhering to PRISMA guidelines ensures transparency and completeness. PROSPERO registration (CRD42023479511) prevents selective reporting. The PRISMA checklist can be referred to in the supplemental material 3 and 4.
- **Comprehensive Search Strategy**: Systematic search across seven major databases ensures broad coverage and reduces publication bias.
- **Clear Eligibility Criteria**: Well-defined criteria for population, interventions, outcomes, settings, and study designs ensure objective study selection. Exclusion of unrelated, duplicated, and non-English studies reduces selection bias.
- **Rigorous Screening Process**: Titles and abstracts screened with discrepancies resolved through consensus, ensuring thorough and unbiased selection.
- **Critical Appraisal Using CASP Tool**: Quality assessment using the CASP tool ensures inclusion of high-quality studies.
- **Detailed Data Extraction and Analysis**: Data extracted in two steps for accuracy and completeness.
- **Quantification of absolute data trends** (e.g. years, diseases, geographical distribution) minimizes bias.
- **Conflict Resolution**: Discrepancies resolved through discussion among authors, ensuring balanced evaluation.

## Discussion

### Implications for future practice and research

Genetic engineering and associated therapies hold significant potential for addressing various human diseases. CRISPR-Cas9, a recent breakthrough, offers a simpler, faster, and more cost-effective approach to genome editing compared to previous methods. However, there are some applications that are presently less prominent, but are inescapable in the future, with respect to the CRISPR-Cas9 system.

Just like the Millennial Prize Problems in mathematics, Surgery has its own unsolved mystery in transplants and the elusive recipient survival. The main root cause of the relatively poor prognosis of transplant recipients remains rejection and infections associated with anti-rejection drugs.

#### Xenotransplantation

Xenotransplantation involving the transfer of animal cells, tissues, or organs to humans, (particularly with pigs as donors), presents a solution to the organ transplant shortage. The key challenge lies in overcoming the issue of trans-species molecular incompatibility, particularly the GAL antigen [87, 88]. Genetic engineering techniques may offer a solution to create rejection-resistant pig organs. Additionally, the potential to design patient-specific organs by incubating human cells in animals could minimize rejection and organ failure risks.

#### Autologous transplantation

The reprogramming of patient-specific cells for transplantation, presents another avenue for medical progress. Lab-grown epidermis [89] is a notable achievement in this field, demonstrating the possibility of producing functional artificial organs using a patient's own stem cells. The transplantation of gene-edited autologous cells involves obtaining somatic cells, converting them into induced pluripotent stem cells (iPSCs), correcting mutations with targeted nucleases, redifferentiating these cells into relevant cell types, and grafting them onto the patient. Human pluripotent stem cells, capable of indefinite self-renewal and differentiation into various cell types, are promising for regenerative repair [90].

While challenges exist in cultivating and transplanting solid organs, such as structural stability and tissue integration, efforts are underway to address these obstacles. The use of 3D printing technology for generating organs is a promising development [91]. Although CRISPR/Cas9 is a relatively new tool in transplantation, its preclinical studies are rapidly expanding, and it may soon revolutionize organ generation.

## Limitations

Limitations of this study include potential oversights due to vague titles, English-only studies, and indexing issues. The review used 82 clinical trials which have been focused on a very narrow spectrum of diseases—where the potential of CRISPR is concerned- that have predominantly been conducted in two countries (The USA and China). This implies that there is a need for more studies with diverse global representations as well as future reviews that represent the everchanging landscape of CRISPR. However, it is imperative to highlight that this review represents one of the most comprehensive analyses on CRISPR, incorporating data from seven distinct databases. Therefore, notwithstanding the outlined limitations, this systematic review significantly contributes to the current body of literature by consolidating and enhancing insights into the utilization of CRISPR in therapeutic clinical trials. Consequently, this review sets a course for the expansion of horizons in gene editing therapy for disease treatment.

## Conclusion

This systematic review consolidated the available clinical trials that utilized CRISPR as a therapeutic agent for genetic diseases. More diversified trials, both in terms of geographical locations and disease classifications, are needed. Ultimately, we hope that this review not only creates awareness about the promising potential of CRISPR-based therapies but also leads to more extensive investigations and collaborations in this groundbreaking field.

The true amalgamation of genetic engineering and regenerative medicine has not yet been achieved. The true potential of this powerful coalition will be unlocked once the key to successfully transform, integrate and proliferate, all cell lines to progenitors, is established. Gene modification and therapy may evolve into synthetic biology, once researchers identify methods to reprogram cell behaviour—thus altering its fate.

In the next 15 years, the landscape of gene editing could look completely different. Genetic engineering, if used wisely, can give us miraculous recoveries. However, malicious use of these mighty technologies is an eminent complication that may lead to production of bioweapons resulting in deadly pandemics.

While some ideas presented above may seem far-fetched at this the moment that could easily change—with CRISPR being accessible and economical. We may live to see a future where modifying genes in all living species, in order to adjust to the environment and the needs of time, may become the norm. The implications of man’s almost divinely interventions, on the course of natural evolution remains to be seen.

Every day science is making progress in such technologies that enable humans to play gods. What remains to be seen is whether this responsibility will prove to be our salvation or annihilation.

## Supplementary Material

iqae013_Supplementary_Data

## Data Availability

Data generated in the present study is presented within the results section of the paper or presented as supplementary material. Appropriate requests can be directed to the corresponding author. This research comprehensively synthesizes clinical trials utilizing CRISPR-Cas9 technology for treating human diseases. It identifies disease targets, intervention types, and advocates for expanded research and collaboration in this transformative field. Overall, the study enriches understanding of CRISPR's therapeutic potential and guides future investigations. This research stands out from others by offering a comprehensive global analysis, including temporal trends as well as future implications and limitations. The findings of this SR prompt the need for high-quality, large, effectiveness-driven randomized controlled trials. Summary of the included studies in the present review. Sickle cell anaemia Thalassaemia beta Biological: Therapeutic vaccine Biological: PD-1 Knockout T Cells Viral Keratitis. Blindness Eye. Herpes Simplex Virus Infection. Cornea Beta-Thalassemia Thalassemia, Genetic Diseases, Inborn, Hematologic Diseases, Hemoglobinopathies Erythrocyte Transfusion Beta-Thalassemia Drug: Luspatercept Other: Placebo Sickle Cell Disease Hydroxyurea Failure, Hydroxyurea Intolerance, Hemoglobinopathies, Hematological Diseases Beta-Thalassemia Thalassemia, Genetic Diseases, Inborn Hematologic Diseases, Hemoglobinopathies Diabetes Mellitus, Diabetes Mellitus, Type 1, Glucose Metabolism Disorders Metabolic Disease, Endocrine System Diseases, Autoimmune Diseases, Immune System Diseases Carcinoma, Non-Small-Cell Lung Metastatic Non Small Cell Lung Cancer Stage IV Non-small Cell Lung Cancer, Squamous Cell Lung Cancer Adenocarcinoma of Lung, Large Cell Lung Cancer B-cell Lymphoma, Non-Hodgkin Lymphoma B-cell Malignancy, Chronic Lymphocytic Leukemia (CLL)/Small Lymphocytic Lymphoma (SLL) Follicular Lymphoma, Mantle Cell Lymphoma Marginal Zone Lymphoma, Large B-cell Lymphoma
