# Supplementary material for: CRISPR CLIP: comprehensive reviews on interventional studies using precision recombinant technologies: clinical landmarks, implications, and prospects
Source: Oxf Open Immunol. 2024 Nov 21;5(1):iqae013. doi: 10.1093/oxfimm/iqae013 (PMC11630829; doi:10.1093/oxfimm/iqae013)
Supplement: iqae013_Supplementary_Data [file iqae013_supplementary_data.zip › Supplementary materials 1 and 2.docx]

| **Detailed search strategy for the CRISPR CLIP systematic review** | | | | | |
| --- | --- | --- | --- | --- | --- |
|  | **Date** | **Host** | **Database/s** | **Search string** | **Results** |
| **1** | 09-Mar-24 | United States National Library of Medicine | Clinicaltrials.gov | (CRISPR OR "Clustered Regularly Interspaced Short Palindromic Repeats") AND (genetic disease OR "genetic disorder" OR "hereditary disease" OR "genetic condition") AND (synthesis OR cure OR treatment OR therapy OR clinical trial)  (CRISPR OR "genome editing" OR "gene editing") AND (genetic disorder OR "inherited disease" OR "genetic mutation") AND (therapy OR treatment OR intervention OR clinical trial)  (CRISPR OR "CRISPR-Cas9" OR "gene editing") AND (genetic condition OR "hereditary disorder" OR "genomic disease") AND (clinical trial OR study OR therapy OR intervention)  (CRISPR OR "gene therapy" OR "genome engineering") AND (genetic disease OR "inherited disorder" OR "DNA mutation") AND (treatment OR cure OR clinical trial OR study) | 102 |
| **2** | 09-Mar-24 | Chinese Clinical Trial Registry | ChiCTR.org.cn |  | 22 |
| **3** | 09-Mar-24 | European Union and EEA | euclinicaltrials.eu and clinicaltrialsregister.eu |  | 12 |
| **4** | 09-Mar-24 | WHO and ICMJE | ISRCTN |  | 1 |
| **5** | 09-Mar-24 | WHO | ICTRP and trialsearch.who.int |  | 79 |
| **6** | 09-Mar-24 | Cochrane | Cochrane |  | 66 |
| **7** | 09-Mar-24 | ICMR's National Institute of Medical Statistics | CTRI |  | 3 |

**Supplementary material 1**

**Supplementary material 2**

| **Information about the 26 studies excluded in the final filtering** | | | | |
| --- | --- | --- | --- | --- |
| **No** | **Study Title** | **Study Type** | **Clinical Trial Number** | **Location** |
| 1 | A clinical study of a novel and highly sensitive protein detection technology based on gold nanoparticles and ssDNA concatemers-activated CRISPR/Cas12a system for diagnosis of esophageal squamous cell carcinoma | Diagnostic test | ChiCTR2200065610 | China |
| 2 | Clinic significance of rapid hypersensitivity detection of Aspergillus fumigatus based on CRISPR | Diagnostic test | ChiCTR2000041176 | China |
| 3 | Clinical study of a novel high sensitivity nucleic acid assay for novel coronavirus pneumonia (COVID-19) based on CRISPR-cas protein | Diagnostic test | ChiCTR2000029810 | China |
| 4 | Establishment a Nucleic Acid Rapid Detection Technology Platform for Detecting Pathogenic Bordetella and Its Drug Resistance Genes | Diagnostic test | ChiCTR2000037764 | China |
| 5 | Rapid identification and clinical transformation of various enterovirus genotypes based on CRISPR technology | Diagnostic test | ChiCTR2000037626 | China |
| 6 | Detection of Enterovirus Genotypes by CRISPR Technology | Observational | NCT04535648 | China |
| 7 | Evaluation of CRISPR-based Test for the Rapid Identification of TB in Pulmonary Tuberculosis Suspects | Observational | NCT04074369 | China |
| 8 | Pathogenic Bordetella Rapid Detection | Observational | NCT04535505 | China |
| 9 | PD-1 Knockout Engineered T Cells for Castration Resistant Prostate Cancer | Observational | NCT02867345 | China |
| 10 | CRISPR study the mechanism of RSPO\WNT in iver fibrosis | Relative factors research | ChiCTR1800014941 | China |
| 11 | The treatment of hybrid exosomes for cartilage defect | No article available | ChiCTR2100041827 | China |
| 12 | Exploiting Epigenome Editing in Kabuki Syndrome: a New Route Towards Gene Therapy for Rare Genetic Disorders | Observational | NCT03855631 | France |
| 13 | Diagnosis of RSTS: Identification of the Acetylation Profiles as Epigenetic Markers for Assessing Causality of CREBBP and EP300 Variants. | Observational | NCT04122742 | France |
| 14 | Characterization of a Functional Test for Mediterranean Family Fever Screening - 2 | Observational | NCT04478409 | France |
| 15 | An Open Label, Single Centric Clinical Trial to assess the Clinical performance of CRISPR-CAS based sensing platform using the BRCA1-Cas12-Strip Assay to detect Breast Cancer - CRISPR-CAS | Observational | CTRI/2023/01/048792 | India |
| 16 | Validation of the TataMD CHECK CRISPR SARS-CoV-2 test 1.0 for the diagnosis of COVID-19 | Observational | CTRI/2021/02/030950 | India |
| 17 | Examining the Knowledge, Attitudes, and Beliefs of Sickle Cell Disease Patients, Parents of Patients With Sickle Cell Disease, and Providers Towards the Integration of CRISPR in Clinical Care | Observational | NCT03167450 | USA |
| 18 | Stem Cells in NF1 Patients With Tumors of the Central Nervous System | Observational | NCT03332030 | USA |
| 19 | A Long-term Follow-up Study in Subjects Who Received CTX001 | Observational | NCT04208529 | USA |
| 20 | A Long-term Follow-up Study of Patients Who Received VOR33 | Observational | NCT05309733 |  |
| 21 | A Clinical Evaluation of Proof Diagnostics Test System Including the Proof Diagnostics Reader and COVID-19 Test | Observational | NCT05331976 |  |
| 22 | Transplacental Transmission of RSV (TTRSV) | Observational | NCT05443607 |  |
| 23 | Epstein-Barr Virus Antibody and Epstein-Barr Virus DNA for Nasopharyngeal Carcinoma Screening | Observational | NCT05447169 |  |
| 24 | Research on the Clinical Characteristics and Key Diagnosis and Treatment Technology of Genetic and Metabolic Liver Disease | Observational | NCT05601557 |  |
| 25 | Novel SARS-CoV-2 Point-of-care Testing | Observational | NCT05034978 |  |
| 26 | A Clinical Evaluation of Pine Trees Health Test System Including the Pine Trees Health Reader and COVID-19 Test for Point-of-Care | Observational | NCT05107258 |  |
